# Work values across generations: Development of the New Work Values Scale (NWVS) and examination of generational differences

**DOI:** 10.3389/fpsyg.2022.1028072

**Published:** 2022-11-07

**Authors:** Barbara Stiglbauer, Marlene Penz, Bernad Batinic

**Affiliations:** Department of Work, Institute of Education and Psychology, Organizational, and Media Psychology, Johannes Kepler University Linz, Linz, Austria

**Keywords:** work values, work orientations, generations, scale development, employer branding

## Abstract

The “battle for talent” requires organizations to more strongly focus on employer branding strategies, and, thus, on work values or work orientations of potential candidates. We therefore developed and validated the *New Work Values Scale* (Study 1; *n* = 316), a brief, 28-item, rating scale that covers a broad set of both, instrumental and symbolic, values, relevant for the appraisal of an employers’ attractiveness. We also applied the scale to a sample representative to the German online population, to explore the controversially discussed generational differences in work values (Study 2; *n* = 956). Results revealed that work values associated with sustainable organizational development or basic needs were highly similar across generations. Younger and older generations only differed significantly with regard to how much they valued clarity, money, career, development, stimulation, and relating, all of which are highly plausible from a lifecycle perspective.

## Introduction

According to a survey by Manpower from the year 2020 (https://go.manpowergroup.com/talent-shortage), the labor market suffers from a dramatic shortage of talents with 75% of all companies reporting hiring difficulties. This represents a sharp increase compared to the last 16 years. The destabilization of the labor market, caused by the pandemic situation due to COVID-19, increases organizational difficulties to recruit well-matching personnel – or even personnel at all. Several sectors currently suffer from high vacancy rates, and new jobs seem to pop up faster than they can be filled (Ramskogler, 2022). This points to the need for organizations to reconsider their recruitment strategies: it is no longer just the job candidate who has to sell him- or herself best to the organization but also the organization which has to sell itself to the candidate. Those kinds of mindsets are nothing new, neither is the awareness of a widespread talent shortage, often termed as “battle for talent” (Beechler and Woodward, 2009). But with the battle for talent expected to further increase in the near future, even those companies that are not having hiring difficulties right now should rethink their strategies for attracting and retaining suitable employees.

Consequently, ‘employer branding’ has been increasingly gaining attention. Even if used in mixed ways, in a very broad definition, employer branding can be understood as all efforts an organization takes to appear as an attractive employer and make people come and stay. Those kinds of efforts might result in a certain image and reputation of an organization (for overview see Theurer et al., 2018). Image and reputation are expected to influence the recruitment process insofar as potential job candidates who agree with a certain image will have higher intentions to apply for a job, accept a job if offered by that organization, or also stay at that organization (Gatewood et al., 1993).

From a potential job candidate’s perspective, a certain organizational image or ‘brand’ is supposed to be the sum of ideas about the organization as an employer or place to work. Those ideas are known to be based on instrumental attributes that serve utilitarian functions (e.g., money, job location, job security, or promotion perspectives). Instrumental attributes are suggested to be the primary constituents of employer branding that serve for the discrimination between a better and a less good employer (Reis et al., 2021). Research further points to the importance of symbolic attributes that serve less utilitarian but rather self-expressive reasons, like innovativeness or prestige. Symbolic attributes are intangible and can be understood as a set of characteristics that form the moral and spirit of an organization (Theurer et al., 2018). Those symbolic attributes appear likewise powerful in predicting applicants’ initial attraction to an organization as place to work and should therefore always be considered (Lievens and Highhouse, 2003).

Instruments measuring instrumental and symbolic attributes within the employer branding framework traditionally focus on the one or the other (Lievens and Slaughter, 2016), suggesting that there is a clear distinction between attributes that serve utilitarian reasons only and those that transport the intangible mindset of the organization as an employer. Lievens (2007) developed an instrument to assess both categories within one scale, but with the very specific focus on the army as an employer. This instrument was further adapted to another very specific occupational context (maritime industry; Rai, 2020). Contradictory with the tradition of the instrumental-symbolic framework, we argue that there is no clear distinction between instrumental and symbolic attributes forming the ‘brand’. We assume that there might be a spill-over from one classification to the other, suggesting the need for an instrument that treats utilitarian and symbolic organizational benefits based on one shared concept, like basic needs and values. One might consider for instance family-supportive arrangements that an organization offers to its employees, such as work time and work location flexibility. Those attributes are utilitarian on the one hand but also symbolic on the other, as they stand for the symbolic organizational attribute to be family friendly. Or, to name a second example, innovation as a symbolic attribute might have instrumental consequences such as to orient oneself in a fast-changing environment.

The aim of the presented work was therefore to develop and validate a context-free, brief rating scale to assess how much a person values those attributes that form the organizational image as an employer or place to work (i.e., *New Work Values Scale*, Study 1). Referring to propositions indicating generational differences in employer branding strategies (Reis and Braga, 2016), we then applied the newly developed scale to a sample representative to the German population, to explore potential generational differences in those values (Study 2).

### Valuable work attributes

Due to the seminal conceptualization by Backhaus and Tikoo (2004), employer branding leads to an organizational image that further influences the perception of the employers’ attractiveness, an important antecedent for the successful recruitment of talents (Backhaus and Tikoo, 2004). A positive employer brand is therefore suggested to be essential to attract and retain the best among available talents (Reis et al., 2021). At the core is the question of those main drivers that motivate people to work for a certain organization. What are the benefits, goods, and mindsets that an organization needs to offer to attract and keep talents? What kind of attributes serve for a differentiation between more and less attractive employers?

Several concepts have been suggested to explain work motivators outside of the employer branding framework, such as work values or work orientations.

**Work values** describe a relatively broad concept of evaluative standards relating to work or the work environment, which can include individual preferences as well as moral standards and social norms (Dose, 1997). This broad definition led to a variety of work value taxonomies and measurement instruments. To overcome the inconsistencies in the work value concept, Consiglio et al. (2017) developed a measure of work values which is grounded in the well-established framework of general life values, the theory of basic personal values by Schwartz (1992). The resulting work values scale is suggested to capture those very fundamental goals people seek in their work life: (1) *Achievement*, (2) *Power*, (3) *Benevolence*, (4) *Universalism*, (5) *Security*, (6) *Tradition*, (7) *Conformity*, (8) *Self-Direction*, (9) *Stimulation*, and (10) *Hedonism* (Consiglio et al., 2017). We consider the allocation of those fundamental goals not only essential for the motivation to work but also an essential basis for the differentiation between a more and a less desired employer. Certainly, with regard to the employer branding framework, more specific aspects regarding a certain job or organization should be considered along with these very fundamental goals. Those job- or organization-specific attributes are more comprehensively considered within the conception of work orientations.

In line with Fossen and Vredenburgh (2014), **work orientations** are work values defined as fundamental purposes paid work serves in the context of one’s life. They understand work orientations as those values an individual seeks to find meaning in a certain job (Fossen and Vredenburgh, 2014). Work orientations are considered as stable traits, conceptualized as tripartite classification of the perception of a job: (1) *Job*, (2) *Career*, and (3) *Calling* (Wrzesniewski et al., 1997). Each one of these work orientations reflects certain feelings and behaviors within the organizational context (Pitacho et al., 2019). Individuals who see their work as a *Job* are more oriented on instrumental attributes, such as monetary rewards. Individuals who face their work as *Career* likewise seek for instrumental, but also symbolic benefits, for instance prestige and status. An attractive employer for individuals who are career-oriented would therefore support career development and advancement. Finally, individuals who hold a *Calling* orientation are expected to work for the pure intrinsic motivation of fulfilment trough work (Pitacho et al., 2019). Willner et al. (2019) extended the established tripartite model by two additional work orientations: (4) *Social-Embeddedness* and (5) *Busyness*. Social-embeddedness-oriented individuals are expected to work mainly for being part of a group or organization, whereas busyness-oriented individuals work to occupy their time (Willner et al., 2019). Interestingly, these work orientations mirror the benefits of work identified in the famous Marienthal study by Marie Jahoda (1981). According to this research, employment not only offers financial benefits (*cf. Job* orientation), but also fulfills basic psychological needs by providing status and identity (*cf. Career* orientation), collective purpose (*cf. Calling* orientation), social contacts (*cf. Social-embeddedness* orientation), as well as activity and time structure (*cf. Busyness* orientation).

Another reevaluation of values in the sense of work orientations was introduced by Höge (2011), who aimed to expand the concept of work orientations with the focus on rather new work realities, namely the work reality of a so called **entreployee**. Entreployees are suggested to work with increased organizational flexibility, self-organization and self-control, reduced hierarchy levels and with high amount of team and project work. Work orientations regarding the entreployee-concept were classified by nine different orientations: the need for (1) *Efficiency*, (2) *Challenge*, (3) *Role Clarity*, (4) *Opportunity Optimizing Career Development*, (5) *Autonomy*, (6) *Security*, (7) *Spatial Flexibility*, (8) *Temporal Flexibility*, and (9) *Segmentation of the Work-Life-Domain* (Höge, 2011). Thus, the entreployee work orientations strongly tap into two of the three basic psychological needs postulated by the Self-Determination Theory (SDT; for overview see Deci et al., 2017): the need for autonomy and the need for competence.^1^

We suggest that all of the reported concepts describing values individuals seek for in the context of work are relevant for the employer branding framework, even if they were seldom considered as theoretical ground in that context. Understanding the values and needs that guide people’s behaviors can be considered as essential for the employer’s brand, as employers will be rated most attractive, when they provide the maximum congruence or fit to those values and needs. Furthermore, person environment fit (PE fit) research has shown that poor person-job and/or person-organization fit is a major predictor of employee withdrawal (e.g., Tak, 2011). Thus, in order to keep their best employees, managers need to know what their employees’ needs and values are (Mitchell et al., 2001). Otherwise, they will leave it to chance whether or not complementary (i.e., employees’ needs are satisfied by what the job/organization is offering) and supplementary fit (i.e., employees and the organization share similar values) will be achieved (*cf.*, van Vianen, 2018).

Standardized and validated measurements are available for all reported concepts. However, to our knowledge, no measure exists that integrates the different approaches. Therefore, the first aim of the present research was to provide a brief measurement that combines all above reviewed aspects into one brief rating scale that can be economically administered in the context of employer branding.

### Generational differences

The question about generational differences in the workplace is a controversially discussed topic. The controversy starts with the definition of a *generation*. In its roots, the idea of a generation was the reference to individuals born within the same historical and socio-cultural context who made comparable formative experiences within a set of historical events they experienced with more or less the same age. As a result, a generation was suggested as a set of individuals who hold to some extent “collective memories” (Lyons and Kuron, 2014). Quantitative research predominantly treats generations at the level of birth-cohorts, with currently four different generations that are represented in the working population. Those cohorts are defined as (1) *Baby Boomers* (born between 1950 and mid-1960s), (2) *Generation X* (born between the early 60s and early or mid-80s), (3) *Generation Y* / *Millennials* (born between mid-80s and late 90s), and (4) *Generation Z* (born between late 80s and late 90s (Lyons and Kuron, 2014; Parry and Urwin, 2011; Pop and Pop, 2019).^2^

Although this “generational approach” has become quite popular, it bears the age-period-cohort problem, as underlying effects might represent either a process of biological aging, specifics of the period when the cohort was observed, or influences associated with a person’s date of birth. In other words, different age groups are at different stages of life, often referred to as “life cycle effect.” Further, individuals are exposed to different events with population-wide effects that vary with the timeframe of assessment. Also, different birth cohorts experience different histories, institutions, and peer-group socialization (Browning et al., 2012). Thus, even if generational differences are observed, their driving forces remain unclear.

Different generations are frequently expected to hold different values and attitudes toward work. This is why generational diversity in organizations might bear the risk for conflict in the work environment and has become an increasing concern at managerial level (Joshi et al., 2011). The issue of generational differences also has been addressed within the scope of employer branding, albeit to a lesser extent. Reis and Braga (2016) for instance reported that economic values were rated with more importance in the context of employer attractiveness with every consecutive generation. In a final consideration they recommend different branding strategies for the respective generation (e.g., positive workplace which encourages creativity for Baby Boomers, development opportunities and good compensation packages for Generation X, and rewards package, development opportunities and positive workplace design for Generation Y).

Besides a general criticism of the research concept of different generations, holding different generational identities (Joshi et al., 2010), there is controversial empirical support for the notion that generations differ in their work values. Reviewing available results regarding this issue, Twenge (2010) for instance comes to the conclusion that work ethic and work centrality (the importance of work in relation to other life domains) declines with every consecutive birth cohort. Intrinsic values, such as finding meaning and interest in work, on the contrary, were relatively consistent across generations. Further, a critical review by Lyons and Kuron (2014) came to the careful conclusion that generations differ in aspects of their work values and attitudes, as well as in leadership and teamwork preferences. Younger generations seem to put more importance on monetary rewards and leisure and are more extroverted, neurotic, and narcissistic. On the other hand, a review addressing generational differences in work values by Parry and Urwin (2011) sums up that empirical evidence is highly inconsistent: many studies fail to find differences and others contradict the popular stereotypes. Similarly, a rather recent review by Cucina et al. (2018) concludes that even if there are significant differences, effect sizes are pretty small.

Within Study 2 we aim to shed more light on this controversial topic by examining generational differences in a broad set of work values within a representative sample of the German population.

## Study 1: Construction of the New Work Values Scale (NWVS)

In line with common recommendations for scale construction (Clark and Watson, 2019; Kyriazos and Stalikas, 2018; Simms, 2008), a literature review on work values and the respective measurement instruments was conducted in a first step (*cf.* Valuable work attributes). Based on this, 15 distinct work values were defined (see Table 1). They cover organizational/cultural attributes which are relevant for supplementary fit (*cf.* Table 1, focus on sustainable organizational development) as well as basic needs and individual motivators which are most important in terms of complementary fit. For the 15 work values an initial item pool was generated by nine individuals (researchers in the field of work and organizational psychology, HR practitioners, employees, and students). Next, a team of four experts reviewed the items, taking conventional criteria regarding content relevancy and formulation into account. The best rated items (six to eight for each defined work value) were included in the validation study and then analyzed in terms of structural characteristics (distributions, exploratory factor analyses EFA, and reliability analyses). This, finally, led to the selection of two items for every work value for the final measurement instrument. Their construct validity was further analyzed by examining the correlations with well-established scales for (entreployee) work orientations (Höge, 2011; Willner et al., 2019) and Schwartz’s theory of basic values (Schmidt et al., 2007).

**Table 1:** Work Values Covered by the New Work Values Scale.

Focus	Work value	Definition: Individuals who value [work value] …
Sustainable organizational development	Readiness for Change	prefer organizations that do not persist in the old, but are open to innovations, consider change as an opportunity, and are ready to implement new ideas.
	Corporate Social Responsibility (CSR)	are looking for organizations that are well aware and take care of their economic, legal, ethical, and philanthropic responsibility.
	Inclusion	want organizations to especially care about fair and respectful treatment of all members and fight discrimination by all means.
Basic needs	Job Security	have a strong need for a secure workplace.
	Participation	prefer a job where hierarchies are flat and everyone is welcome to contribute his or her own opinion and ideas.
	Clarity	need structure, rules, and guidelines that provide stability, consistency, and orientation.
	Flexibility	want their job not to interfere with their personal lives.
Individual motivators	Money	are mostly motivated by monetary rewards.
	Career	consider career development opportunities as very important to them.
	Development	want to always give their best and to therefore further develop their professional knowledge, skills, and competencies.
	Stimulation	have a strong need for variety, challenges, and much going on in their job.
	Autonomy	want to design and do their work self-directedly.
	Meaning	need their work to be meaningful and serve a collective purpose.
	Relating	place great emphasis on good social relationships at work.
	*Comfort**^1^*	*want their job to be a feel-good-place where they do not feel pressured or stressed.*

1 Comfort was not included in the final measurement instrument, as the data did not provide evidence for a distinct factor.

### Materials and methods

#### Participants and procedure

Three hundred and thirty individuals completed an online questionnaire including questions regarding demographic and employment-related characteristics, the newly developed items, and three well-established scales for the construct validity analyses. They were randomly selected from the German population of *Respondi*’s (www.respondi.com) online access panel members and received bonus points for their participation that they could eventually swap for products. Excluding respondents with unreasonable response times led to a final sample of *n* = 316 individuals (57.6% female; 41.1% male; 0.3% other), aged 18 to 65 years (*M* = 45.62, *SD* = 13.78) with levels of education ranging from compulsory (14.9%) to university (23.4%) levels (37.0% vocational and 23.7% high school diploma). The majority (66.8%) was employed, 9.2% were in education, and the remaining participants were unemployed/out of the labor force.

#### Measures

The 97 newly developed work values items (six to eight per work value) that were included in this study are reported in Table A2 in the Supplementary material (please note that the items were developed in German language; however, English translations can be found in Table A2 as well). To examine construct validity of the newly developed scale, three well-established measures were included (their reliability estimates are reported in Table A3 in the Supplementary material): First, Willner et al.’ (2019) Work Orientation Questionnaire assesses the orientations *Job*, *Career*, *Calling*, *Social-Embeddedness*, and *Busyness* with five items each on a 7-point response scale (1 = *not at all*; 7 *very much*). Second, the Entreployee Work Orientation Scales (Höge, 2011) assess the nine needs relevant in the entreployee context defined by Höge (2011) with 25 items in total using a 6-point response format with 1 = *unimportant* and 6 = *very important*. Third, the German 21-item version of the Portraits Value Questionnaire (PVQ; Schmidt et al., 2007) was used to measure the ten basic values of Schwartz’s theory with two items each (and three items for universalism) on a 6-point response scale (1 *= very much like me*; 6 = *not like me at all*).

### Results

The factor loadings of the EFA including the most relevant two items for every work value are shown in Table 2. The work value *Comfort* and its associated items were excluded due to substantial overlap with other work values. Variance explained by the 14 factors was 62.74%. The items loaded on the expected factor, and most items demonstrated negligible cross-loadings. Reliability estimates were mostly acceptable to good (*cf.* Table 2).

**Table 2:** Item Loadings (EFA, Promax Rotation, Structure Matrix) and Spearman Brown Reliability Estimates for the 14 Work Values.

Item	RFC	CSR	INC	SEC	PAR	CLA	FLE	MON	CAR	DEV	STI	AUT	MEA	REL
rfc1	**0.85**	0.21	0.18	0.11	0.27	0.20	0.21	0.03	0.23	0.24	0.28	0.16	0.09	0.15
rfc2	**0.61**	0.38	0.35	0.05	0.29	0.15	0.20	0.13	0.34	0.35	0.26	0.22	0.20	0.20
csr1	0.23	**0.83**	0.45	0.01	0.36	0.04	0.16	−0.13	0.01	0.24	0.20	0.15	0.52	0.24
csr2	0.28	**0.77**	0.41	0.04	0.36	0.13	0.17	−0.03	0.05	0.25	0.16	0.06	0.39	0.17
inc1	0.22	0.44	**0.88**	0.20	0.31	0.18	0.18	0.04	0.14	0.23	0.18	0.17	0.39	0.28
inc2	0.27	0.35	**0.48**	0.10	0.15	0.02	0.11	0.12	0.07	0.18	0.13	0.07	0.31	0.26
sec1	0.12	0.01	0.23	**0.84**	0.19	0.21	0.13	0.19	0.10	0.14	0.05	0.01	0.06	0.26
sec2	0.08	0.02	0.17	**0.86**	0.13	0.29	0.28	0.21	0.14	0.12	0.03	0.05	0.09	0.22
par1	0.26	0.33	0.25	0.10	**0.78**	0.16	0.32	−0.04	0.12	0.19	0.25	0.20	0.19	0.12
par2	0.26	0.35	0.26	0.13	**0.76**	0.09	0.18	−0.05	0.05	0.16	0.13	0.19	0.22	0.20
cla1	0.16	0.07	0.12	0.21	0.18	**0.68**	0.24	0.07	0.13	0.19	0.14	0.05	0.11	0.00
cla2	0.19	0.08	0.16	0.25	0.13	**0.85**	0.19	0.15	0.11	0.17	0.13	−0.04	0.13	−0.01
fle1	0.19	0.16	0.16	0.14	0.26	0.16	**0.69**	0.07	0.11	0.11	0.07	0.27	0.12	0.28
fle2	0.09	0.14	0.23	0.22	0.44	0.22	**0.42**	0.11	0.05	0.12	0.08	0.20	0.12	0.12
mon1	0.09	−0.16	0.00	0.23	−0.05	0.07	0.12	**0.79**	0.30	0.02	0.03	0.20	−0.06	0.07
mon2	0.03	−0.02	0.09	0.13	−0.02	0.14	0.07	**0.77**	0.25	0.08	0.05	0.09	−0.01	0.09
car1	0.29	0.10	0.19	0.12	0.17	0.15	0.17	0.28	**0.84**	0.57	0.47	0.27	0.19	0.33
car2	0.28	−0.02	0.09	0.15	0.06	0.08	0.18	0.32	**0.82**	0.51	0.38	0.26	0.18	0.19
dev1	0.24	0.23	0.19	0.10	0.13	0.14	0.13	0.07	0.54	**0.86**	0.44	0.17	0.33	0.26
dev2	0.36	0.24	0.20	0.14	0.29	0.23	0.23	0.04	0.62	**0.85**	0.51	0.32	0.41	0.24
sti1	0.26	0.15	0.17	0.02	0.18	0.09	0.08	0.05	0.32	0.33	**0.74**	0.29	0.23	0.23
sti2	0.31	0.21	0.13	0.06	0.23	0.18	0.15	0.03	0.56	0.60	**0.80**	0.26	0.37	0.13
aut1	0.17	0.14	0.14	0.04	0.26	0.02	0.27	0.17	0.27	0.21	0.32	**0.81**	0.24	0.18
aut2	0.21	0.07	0.14	0.04	0.16	0.01	0.32	0.10	0.27	0.26	0.27	**0.69**	0.21	0.06
mea1	0.13	0.44	0.45	0.12	0.31	0.17	0.11	−0.03	0.17	0.40	0.31	0.22	**0.78**	0.30
mea2	0.15	0.53	0.38	0.03	0.21	0.09	0.17	−0.04	0.17	0.35	0.30	0.24	**0.94**	0.32
rel1	0.14	0.15	0.24	0.21	0.12	0.00	0.22	0.10	0.23	0.21	0.17	0.14	0.25	**0.72**
rel2	0.26	0.24	0.27	0.23	0.26	0.06	0.39	0.10	0.29	0.40	0.33	0.14	0.32	**0.70**
Reliability	0.66	0.77	0.58	0.82	0.73	0.74	0.46	0.74	0.80	83	0.71	0.71	0.84	0.66

RFC = Readiness for Change, CSR = Corporate Social Responsibility, INC = Inclusion, SEC = Security, PAR = Participation, CLA = Clarity, FLE = Flexibility, MON = Money, CAR = Career, DEV = Development, STI = Stimulation, AUT = Autonomy, MEA = Meaning, REL = Relating. Bold values indicate loadings on the proposed factor.

Correlations of the 14 work values provided by the NWVS with the constructs of the three other well-established measurement instruments (Work Orientation Questionnaire, WOQ, Willner et al. 2019; Entreployee Work Orientation Scale, EWOS, Höge, 2011; and the Portraits Value Questionnaire, PVQ, Schmidt et al., 2007) were largely as expected (see Table A1 and A3 in the Supplementary material for construct definitions and detailed results) and support construct validity of the NWVS: *Corporate Social Responsibility (CSR)* and *Inclusion* correlated most strongly with *Universalism* (PVQ); *Job Security* related most strongly to the value *Security* (PVQ) and the *Need for Security* (EWOS). *Clarity* and *Flexibility* demonstrated the highest correlations with the *Need for Clarity* (EWOS) and the *Needs for Spatial* or *Temporal Flexibility* and *Segmentation* (EWOS), respectively. *Money* related most strongly with *Power* (PVQ) and *Job Orientation* (WOQ)*. Career* revealed the highest relationships with *Achievement and Power* (PVQ), *Career Orientation* (WOQ) and the *Need for Career Development* (EWOS). *Development* most strongly related to *Career Orientation* (WOQ) as well as the *Needs for Efficiency* (EWOS) and *Achievement* (PVQ)*. Stimulation*/*Autonomy* had their highest correlations with the respective values (*Stimulation*/*Self-Direction;* PVQ) and needs (*Challenge*/*Autonomy;* EWOS). Also, in line with expectations, *Meaning* correlated highly with the *Calling Orientation* (WOQ), while *Relating* correlated most strongly with *Social-Embeddedness Orientation* (WOQ). Additionally, there were significant, but not as high correlations between *Participation* and the values *Self-Direction*, *Universalism*, and *Benevolence* (PVQ). *Readiness for Change* was expected to most strongly relate to low levels of the value of *Tradition* (PVQ), which, however, could not be confirmed by the results. Thus, this work value seems to capture aspects other than traditional values.

## Study 2: Generational differences in work values

In a next step, the NWVS was administered to a sample representative to the German online population with the aim to examine potential generational differences in work values.

### Materials and methods

#### Participants and procedure

The representative German online sample was recruited with the help of the panel provider *Respondi* (www.respondi.com). As in Study 1, respondents (*N* = 1.115) received bonus points for completing the short online questionnaire that included socio-demographics and employment-related characteristics as well as the newly developed work values scale. Participants with very short response times and who were no longer part of the working population (i.e., retirees) were excluded, resulting in a final sample of *n* = 956 (47.3% female; 52.3% male; 0.4% other), aged 16 to 65 years (*M* = 41.92, *SD* = 13.51) and with different levels of education (24.8% compulsory, 26.3% vocational, 20.6% high school, and 28.3% university). Three quarters (75.2%) were employed (full-time, part-time, or self-employed), 11.0% were in education, and 13.8% unemployed/out of the labor force.

#### Measures

Work values were measured with the newly developed NWVS (Table A4 in the Supplementary material reports the final scale). Responses were scored on a 5-point scale with 1 = *do not agree at all*, 3 = *part-part*, and 5 = *fully agree*. A Confirmatory Factor Analysis (CFA) with the 14 work values modelled as latent constructs indicated by the respective two items provided good support for the proposed factor structure, Χ^2^(259) = 734.18, *p* < 0.001, Comparative Fit Index (CFI) = 0.957, Tucker Lewis Index (TLI) = 0.932, and Root Mean Square Error of Approximation (RSMEA) = 0.044. Factor loadings ranged from 0.63 to 0.89, and Spearman Brown reliability estimates from 0.67 to 0.87 (see Table A5 in the Supplementary material for detailed results).

Generations were defined based on respondents’ age, relying on conventional classifications (*cf.* Eberhardt, 2021) of *Babyboomers* comprising the birth cohorts 1950 to 1964 (19.8%), *Generation X* the cohorts 1965 to 1979 (30.2%), *Generation Y* the cohorts 1980 to 1994 (32.8%), and *Generation Z* the cohorts 1995 to 2009 (17.2%).

### Results

To examine generational differences in work values based on the NWVS, a multivariate analysis of covariances (MANCOVA) was conducted with the 14 work values as dependent variables and generation as the independent variable. To control for potential confounding, gender (two dummy variables representing *female* and *divers*), education (continuous variable from 1 representing the lowest level of education, i.e., compulsory, to 4 representing the highest level, i.e., university), and employment status (three dummy variables indicating *unemployed*, *out of the labor force*, and *in education*) were included as covariates (*cf.* Table A6 in the Supplementary material, which reports the bivariate correlations of the work values with sociodemographic characteristics).

**Figure 1:**
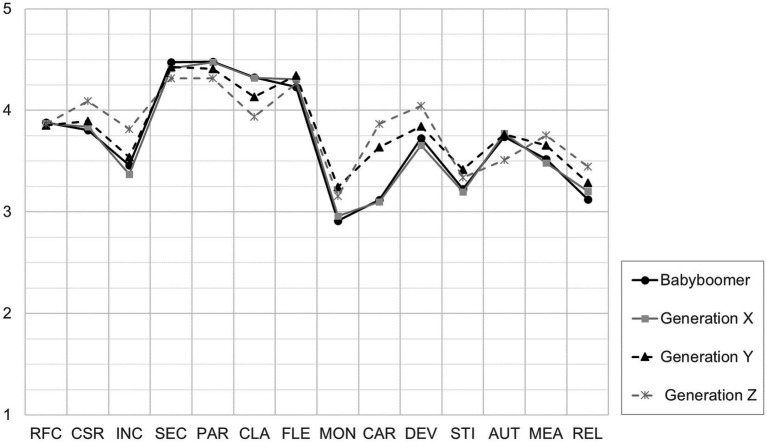
Mean Work Values of the Generations. RFC = Readiness for Change, CSR = Corporate Social Responsibility, INC = Inclusion, SEC = Security, PAR = Participation, CLA = Clarity, FLE = Flexibility, MON = Money, CAR = Career, DEV = Development, STI = Stimulation, AUT = Autonomy, MEA = Meaning, REL = Relating.

Results showed a significant multivariate effect of generation, revealing that, overall, younger cohorts hold stronger work values, *F*(42, 2,805) = 3.66, *p* < 0.001, part. η^2^ = 0.052. Significant univariate effects were found for six out of the 14 work values: clarity, money, career, development, stimulation, and relating (*cf.* Table 3). Post-hoc, Bonferroni corrected, multiple comparisons (*cf.* Table 3) revealed that the differences were primarily between the two older cohorts (putting higher emphasis on *Clarity*) and the two younger cohorts (holding stronger values towards *Money*, *Career*, and *Stimulation*). Generation Z also hold significantly stronger values towards *Development* and *Relating* than Generation X. Figure 1 illustrates the mean work values for each generation.

**Table 3:** Generational Differences in Work Values (Univariate Effects).

	MANCOVA: Univariate effects			Descriptive statistics: *M* (*SD*)				Significant differences (Bonferroni corrected; *p* < 0.05)
Work value	*F* (3, 946)	*p*	part. η^2^	Baby-boomer (B)	Generation X	Generation Y	Generation Z	
RFC	0.33	0.803	0.001	3.88 (0.83)	3.87 (0.81)	3.85 (0.83)	3.87 (0.87)	
CSR	0.45	0.718	0.001	3.80 (1.07)	3.84 (0.96)	3.90 (0.85)	4.09 (0.93)	
INC	1.71	0.163	0.005	3.46 (1.05)	3.37 (1.02)	3.54 (0.93)	3.81 (0.94)	
SEC	0.26	0.854	0.001	4.47 (0.78)	4.41 (0.80)	4.43 (0.75)	4.32 (0.87)	
PAR	1.87	0.132	0.006	4.48 (0.66)	4.47 (0.68)	4.41 (0.64)	4.31 (0.82)	
CLA	4.74	0.003	0.015	4.32 (0.74)	4.32 (0.69)	4.14 (0.71)	3.94 (0.80)	B-Z; X-Y; X-Z
FLE	0.64	0.592	0.002	4.23 (0.70)	4.31 (0.70)	4.35 (0.67)	4.26 (0.80)	
MON	11.64	0.000	0.036	2.91 (0.93)	2.96 (1.05)	3.25 (0.97)	3.16 (0.90)	B-Y; B-Z; X-Y; X-Z
CAR	25.79	0.000	0.076	3.12 (0.98)	3.10 (1.07)	3.64 (0.98)	3.87 (0.98)	B-Y; B-Z; X-Y; X-Z
DEV	3.51	0.015	0.011	3.72 (0.91)	3.65 (1.00)	3.84 (0.89)	4.05 (0.81)	X-Z
STI	5.39	0.001	0.017	3.22 (0.92)	3.20 (0.97)	3.42 (0.89)	3.34 (0.93)	X-Y; X-Z
AUT	1.14	0.330	0.004	3.74 (0.86)	3.78 (0.92)	3.76 (0.82)	3.51 (0.88)	
MEA	0.06	0.983	0.000	3.52 (0.95)	3.48 (1.09)	3.66 (0.92)	3.75 (0.98)	
REL	3.95	0.008	0.012	3.12 (0.88)	3.2 (0.95)	3.29 (0.91)	3.45 (0.81)	B-Z; X-Z

RFC = Readiness for Change, CSR = Corporate Social Responsibility, INC = Inclusion, SEC = Security, PAR = Participation, CLA = Clarity, FLE = Flexibility, MON = Money, CAR = Career, DEV = Development, STI = Stimulation, AUT = Autonomy, MEA = Meaning, REL = Relating.

## Discussion

The present research was conducted within a context of dramatic deterioration in talent shortage and the resulting need for organizations to optimize their branding strategies, summarized by the term “employer branding.” Traditionally, employer branding has focused either on so called instrumental (e.g., pay and benefits) or symbolic (e.g., maintaining self-identity, enhancing someone’s self-image) attributes associated with the attractiveness of an organization as place to work. Consequently, scales measuring attributes in the context of employer branding rather focused on the one or the other (Lievens, 2007; Lievens and Slaughter, 2016). With Study 1, we aimed to provide a brief and validated measurement for the rating of a broad set of both, instrumental and symbolic, values, associated with the appraisal of an employers’ attractiveness, the New Work Values Scale (NWVS). With Study 2, we further investigated whether different generations hold different work values and should therefore be addressed in different ways within employer branding strategies.

### Construction of the NWVS

The theoretical ground for our selection of the respective set of work values are well established need and value theories, namely the theory of basic personal work values (Consiglio et al., 2017; Schwartz, 1992), the seminal concept of work orientations (Wrzesniewski et al., 1997) including current extensions and work trends by Willner et al. (2019) and Höge (2011), and the framework of self-determination theory (Deci et al., 2017; van den Broeck et al., 2016). The NWVS is therefore unique in the embracement of different value theories, assessed with a convenient number of items (28 items encompassing 14 dimensions) and brief processing time (response times of five to ten minutes). Abessolo et al. (2021) recently used a similar approach by combining common frameworks regarding work attributes (work values, work orientations, and career anchors) into one validated measurement. However, the resulting questionnaire claims to measure career choices across the life span and not work-related values in general. Furthermore, Abessolo et al. (2021) looked for overlaps between the selected frameworks, whereas our claim was to integrate the different, additional aspects of several sound value concepts. As a consequence, the NWVS shows a broader set of underlying dimensions. Furthermore, the 15 work values covered by the NWVS include attributes relevant for both supplementary and complementary person-job/organization fit (van Vianen, 2018).

Reliability analyses and exploratory as well as confirmatory factor analyses provide strong support for the 14-factor structure of the NWVS. All 28 items loaded on the expected factors and showed negligible cross-loadings as well as acceptable to good reliability estimates. Construct validity was also supported by correlational analyses between the NWVS and well-established measures. Taken together, the NWVS can be considered a sound instrument to briefly assess a wide range of different work values.

### Generational differences

During the last decade there has been an increase in literature discussing generational differences in work values, mainly with the focus on managerial concerns. Currently, the entrance of post-millennials, also called Generation Z, into the labor market, seems to create some tension, assuming that this young work generation will differ significantly from precedent generations. For instance, it is assumed that generation Z will behave more narcissistic, mentally instable, achievement-oriented, and socially interconnected than their predecessors (Schroth, 2019). Generational differences in the workplace hit the popular press as well as empirical research, but with divergent conclusions. Consulting popular press gives the impression that generational differences in the work environment are axiomatic and have to result in new leadership guidance. On the contrary, empirical evidence for the stated differences seems to be weak and/or divergent (Rudolph et al., 2018). Against this background we decided to test for generational differences in the 14 work values assessed by the NWVS. We found significant differences in only 6 out of these 14 work values:

- *Clarity*: having a strong need for structure, rules, and guidelines that provide stability, consistency, and orientation
- *Money*: being motivated by monetary rewards
- *Career*: considering career development opportunities as very important
- *Development*: always striving to be at the best and to therefore develop knowledge, skills, and competencies
- *Stimulation*: wanting variety, challenges, and much going on
- *Relating*: placing great emphasis on good social relationships

Thus, work values associated with sustainable organizational development (i.e., *Readiness for Change*, *CSR*, and *Inclusion*), basic needs (except for *Clarity*; i.e., *Security*, *Participation*, and *Flexibility*) as well as highly intrinsic aspects (i.e., *Autonomy* and *Meaning*) were pretty similar across different generations. The main generational differences became apparent in individual motivators only and here between the two older compared to the two younger cohorts. Baby boomers and Generation X valued *Clarity* more than did Generation Y and Z, whereas Generation Y and Z reported stronger values towards *Money*, *Career*, and *Stimulation* compared to their predecessors. Additionally, results showed a significant difference between Generation Z and Generation X in regard to *Development* and *Relating*, with Generation Z putting more emphasis on these two work values.

It seems not surprising that the youngest work generation holds the strongest emphasis on *Development*, considering that they are in the early beginning of their careers. Similarly, the high emphasis of Generation Z towards *Relating* might represent an age-related preference for intense social exchange, that might decrease as soon as people feel settled in their family and social lives. In general, our results can be considered as supportive for the theory of life-stages or life-cycles rather than the assumption of significantly distinct generational work identities (*cf.* Joshi et al., 2010).

Interestingly, overall, all generations put a relatively low emphasis on money as the key motivator to work. Although this might appear surprising at a first glance, it is in line with the idea of Jahoda’s latent benefits of work. According to Jahoda (1981), important drivers why people seek to work are symbolic attributes, such as time structure, social experiences, and personal identity and growth. Stiglbauer and Batinic (2012) could demonstrate that the amount to which employers provide access to these latent benefits significantly contributes to employee’s commitment to work. Transferring this rationale to the context of employer branding, a good salary might be important but not sufficient to motivate people to work for a certain organization. The distinction between a “good” and “not so good” employer or place to work may be based on other factors, such as the amount of appreciation in terms of involvement and flexibility an organization shows to its employees. In its theoretical implication, this notion is in line with Herzberg’s seminal two-factor theory of work motivation, which claims that money, defined as hygiene factor, has the potential to prevent job-dissatisfaction and causes dissatisfaction if not adequately provided. On the contrary, monetary rewards do not significantly contribute to satisfaction and work motivation (for an overview about Herzberg’s theory, e.g., Alshmemri et al., 2017). Or, put into other words, money just matters, if not satisfied.

### Limitations, future directions, and implications

As the NWVS is a newly developed scale, of course more research is needed to further prove its validity. Another limitation might be represented by the fact that data collection took place in times of COVID-19 pandemic, which affected the labor market at a great pace. It can be hypothesized that this radical change also influenced participant’s work values that might have been rated differently before the labor market shock. Consequently, we consider our results representative for the current work situation, which is a post pandemic situation. Future research will be needed to explore situational differences vs. stability in employees’ work values based on the NWVS. We further want to mention that the NWVS solely considers the perspective of an employee or future job candidate by neglecting the perspective of the organization. It might represent a promising future project to work on a complementary tool, which addresses those attributes that an organization considers as important and essential for creating the organizational brand and attract the ideal job candidates.

However, we would like to highlight that the NWVS is unique in its conception and should provide high practical value in the context of employer branding. Major advantages are the short assessment time and the broad coverage of work values that all have been shown to significantly contribute to the attractiveness of an employer’s brand. The NWVS can therefore be used by companies who consider to reevaluate their strategies to attract talents and/or improve the retention of already hired personnel, for example by implementing benefits and conveniences suited to the desires of their members. Furthermore, it can be used as convenient research instrument in the context of employer branding and employer attractiveness. In addition to this, the NWVS is also suitable for questions relating to person-organization fit. Tanwar and Kumar (2019) state that value-based employer brand dimensions (e.g., positive work culture, corporate social responsibility, or salary) help for the creation of a person-organization-fit. Person-organization fit has been increasingly considered as “extra-role behavior” in personnel selection processes. It is concluded that the congruence in certain values that employees experience with their employers (and vice versa) regulates the selection process as well as positive outcomes, such as climate for well-being, low levels of turnover and absenteeism, and cooperation (Morley, 2007; Schneider, 2001). Congruence in values between an employee and its employer was further shown to significantly contribute to job satisfaction, organizational commitment, and intention to remain in a certain job (Westerman and Cyr, 2004). The NWVS can therefore help to find the ideal organization that fits best with individual preferences and can be considered as a low-threshold career counseling tool.

Having applied the NWVS to different generations, we agree with several other empirical examinations that failed to find considerable generational differences in regard to work attributes, preferences, and values (reviews, e.g., Cucina et al., 2018; Lyons and Kuron, 2014; Parry and Urwin, 2011; Rudolph et al., 2018). At the same time, we would like to point out that the lack of substantial generational differences in work values does not prove the absence of those differences. Originally emerged from the field of sociology, the theory of distinguishable generations, that hold respective shared collective memories and identities, was not conceptualized to be tested at quantitative level. As a consequence, quantitative research regarding generational differences is hypothesized to be flawed by imprecise definitions (and operationalizations of those definitions) of what exactly is expected from a certain generation to be unique (Rudolph et al., 2018). Results of studies on generational differences are further confounded by effects that might rather be due to age (maturation effects) or a certain period or timeframe, when a certain measurement was provided (period effects). Those confounds are referred to as age-period-cohort problem (Browning et al., 2012; Parry and Urwin, 2011), and, of course, also are true for our research.

Overall, our research supports the notion that *Babyboomers*, *Generation X*, *Generation Y*, and *Generation Z* do not differ as much in their work values as postulated by popular media. Particularly, as long as work values refer to sustainable organizational development, basic needs, and highly intrinsic aspects, the generations seem to be highly similar. And the fact that younger as compared to older generations are more strongly motivated by *Money*, *Career*, and *Development*, or also by *Stimulation* and *Relating*, is highly plausible from a lifecycle perspective. For organizations, we therefore highly recommend to adopt a lifecycle perspective to attract and retain employees.

## Data availability statement

The raw data supporting the conclusions of this article will be made available by the authors, without undue reservation.

## Ethics statement

Ethical review and approval was not required for the study on human participants in accordance with the local legislation and institutional requirements. Written informed consent from the participants’ legal guardian/next of kin was not required to participate in this study in accordance with the national legislation and the institutional requirements.

## Author contributions

BS and BB contributed to conception and design of the study and organized the database. BS performed the statistical analyses. BS and MP wrote the first draft and sections of the manuscript. All authors contributed to manuscript revision, read, and approved the submitted version.

## Funding

The Johannes Kepler University Linz offered funding for open access publication fees.

## Conflict of interest

The authors declare that the research was conducted in the absence of any commercial or financial relationships that could be construed as a potential conflict of interest.

## Publisher’s note

All claims expressed in this article are solely those of the authors and do not necessarily represent those of their affiliated organizations, or those of the publisher, the editors and the reviewers. Any product that may be evaluated in this article, or claim that may be made by its manufacturer, is not guaranteed or endorsed by the publisher.

## Footnotes

^1^The SDT is a macro theory of human motivation that has successfully been applied to the context of work motivation. It postulates three basic psychological needs: the need for autonomy, competence and relatedness that, when satisfied, promote motivation, wellbeing, and effective performance (Deci et al., 2017). Based on SDT, employers should support their employees to gain competencies and feel confident, provide freedom in the work processes to allow to develop and live their own working behaviors, and provide respect and belonging (Deci et al., 2017). Van den Broeck et al. (2016) recommend to apply SDT to organizational thinking, as competence, autonomy and relatedness are considered to improve the work environment if addressed adequately.

^2^Please note that there is no uniform definition of the cohorts, and, depending on the authors, the classification of the cohorts differs by a few years.

## References

[ref1] Abessolo M. Hirschi A. Rossier J. (2021). Development and validation of a multidimensional career values questionnaire: a measure integrating work values, career orientations, and career anchors. J. Career Dev. 48, 243–259. doi: 10.1177/0894845319846567

[ref2] Alshmemri M. Shahwan-Akl L. Maude P. (2017). Herzberg’s two-factor theory. Life Science Journal 14, 12–16. doi: 10.7537/marslsj140517.03

[ref3] Backhaus K. Tikoo S. (2004). Conceptualizing and researching employer branding. Career Dev. Int. 9, 501–517. doi: 10.1108/13620430410550754

[ref4] Beechler S. Woodward I. C. (2009). The global war for talent. J. Int. Manag. 15, 273–285. doi: 10.1016/j.intman.2009.01.002

[ref5] Browning M. Crawford I. Knoef M. (2012). The age-period cohort problem: set identification and point identification (cemmap working paper No. CWP02/12). Available at http://hdl.handle.net/10419/64757

[ref6] Clark L. A. Watson D. (2019). Constructing validity: new developments in creating objective measuring instruments. Psychol. Assess. 31, 1412–1427. doi: 10.1037/pas0000626, PMID: 30896212 PMC6754793

[ref7] Consiglio C. Cenciotti R. Borgogni L. Alessandri G. Schwartz S. H. (2017). The WVal: a new measure of work values. J. Career Assess. 25, 405–422. doi: 10.1177/1069072716639691

[ref8] Cucina J. M. Byle K. A. Martin N. R. Peyton S. T. Gast I. F. (2018). Generational differences in workplace attitudes and job satisfaction: lack of sizable differences across cohorts. J. Manag. Psychol. 33, 246–264. doi: 10.1108/JMP-03-2017-0115

[ref9] Deci E. L. Olafsen A. H. Ryan R. M. (2017). Self-determination theory in work organizations: the state of a science. Annu. Rev. Organ. Psych. Organ. Behav. 4, 19–43. doi: 10.1146/annurev-orgpsych-032516-113108

[ref10] Dose J. J. (1997). Work values: an integrative framework and illustrative application to organizational socialization. J. Occup. Organ. Psychol. 70, 219–240. doi: 10.1111/j.2044-8325.1997.tb00645.x

[ref11] Eberhardt D. (ed.) (2021). Generationen zusammen führen. Mit generation X, Y, Z und Babyboomern die Arbeitswelt gestalten [Bringing generations together. Shaping the world of work with generation X, Y, Z and baby boomers]. Freiburg: Haufe.

[ref12] Gatewood R. D. Gowan M. A. Lautenschlager G. J. (1993). Corporate image, recruitment image and initial job choice decisions. Acad. Manag. J. 36, 414–427. doi: 10.2307/256530

[ref13] Fossen R. J. S.-V. Vredenburgh D. J. (2014). Exploring differences in work’s meaning: an investigation of individual attributes associated with work orientations. J. Behav. Appl. Manag. 15, 101–120. doi: 10.21818/001c.17940

[ref14] Höge T. (2011). Perceived flexibility requirements at work and the entreployee-work-orientation: concept and measurement. Innsbruck Journal Psychologie des Alltagshandelns / Psychology of Everyday Activity 4, 3–21.

[ref15] Jahoda M. (1981). Work, employment, and unemployment: values, theories, and approaches in social research. Am. Psychol. 36, 184–191. doi: 10.1037/0003-066X.36.2.184

[ref16] Joshi A. Dencker J. C. Franz G. (2011). Generations in organizations. Res. Organ. Behav. 31, 177–205. doi: 10.1016/j.riob.2011.10.002

[ref17] Joshi A. Dencker J. C. Franz G. Martocchio J. J. (2010). Unpacking generational identities in organizations. Acad. Manag. Rev. 35, 392–414. doi: 10.5465/AMR.2010.51141800

[ref18] Kyriazos T. A. Stalikas A. (2018). Applied psychometrics: the steps of scale development and standardization process. Psychology 09, 2531–2560. doi: 10.4236/psych.2018.911145

[ref19] Lievens F. Highhouse S. (2003). The relation of instrumental and symbolic attributes to a company’s attractiveness as an employer. Pers. Psychol. 56, 75–102. doi: 10.1111/j.1744-6570.2003.tb00144.x

[ref20] Lievens F. (2007). Employer branding in the Belgian army: the importance of instrumental and symbolic beliefs for potential applicants, actual applicants, and military employees. Hum. Resour. Manag. 46, 51–69. doi: 10.1002/hrm.20145

[ref21] Lievens F. Slaughter J. E. (2016). Employer image and employer branding: what we know and what we need to know. Annu. Rev. Organ. Psych. Organ. Behav. 3, 407–440. doi: 10.1146/annurev-orgpsych-041015-062501

[ref22] Lyons S. Kuron L. (2014). Generational differences in the workplace: a review of the evidence and directions for future research. J. Organ. Behav. 35, S139–S157. doi: 10.1002/job.1913

[ref23] Mitchell T. R. Holtom B. C. Lee T. W. (2001). How to keep your best employees: developing an effective retention policy. Acad. Manag. Exec. 15, 96–108. doi: 10.5465/ame.2001.5897929

[ref24] Morley M. J. (2007). Person-organization fit. J. Manag. Psychol. 22, 109–117. doi: 10.1108/02683940710726375

[ref25] Parry E. Urwin P. (2011). Generational differences in work values: a review of theory and evidence. Int. J. Manag. Rev. 13, 79–96. doi: 10.1111/j.1468-2370.2010.00285.x

[ref26] Pitacho L. A. Palma P. Correia P. (2019). Work orientation: dimensionality and internal model. Analise Psicologica 37, 479–491. doi: 10.14417/ap.1667

[ref27] Pop D. Pop M. T. (2019). Approaching the labor market from a generational perspective. IOP Conference Series: Materials Science and Engineering 568:012084. doi: 10.1088/1757-899X/568/1/012084

[ref28] Rai A. (2020). An application of the instrumental-symbolic framework in maritime industry: a study on employer branding among seafarers. Manag. Res. Rev. 43, 270–292. doi: 10.1108/MRR-04-2019-0181

[ref29] Ramskogler P. (2022). “Feeling the heat? Assessing labor shortages in the euro area,” SURF Policy Brief, 266. Available at https://www.suerf.org/suer-policy-brief/39585/feeling-the-heat-assessing-labor-shortages-in-the-euro-area

[ref30] Reis G. G. Braga B. M. (2016). Employer attractiveness from a generation perspective: implications for employer branding. Revista de Administração 51, 103–116. doi: 10.5700/rausp1226

[ref31] Reis I. Sousa M. J. Dionisio A. (2021). Employer branding as a talent management tool: a systematic literature revision. Sustainability 13:10698. doi: 10.3390/su131910698

[ref32] Rudolph C. W. Rauvola R. S. Zacher H. (2018). Leadership and generations at work: a critical review. Leadership Quarterly 29, 44–57. doi: 10.1016/j.leaqua.2017.09.004

[ref33] Schmidt P. Bamberg S. Davidov E. Herrmann J. Schwartz S. H. (2007). Die Messung von Werten mit dem Portraits Value Questionnaire. Z. Sozialpsychol. 38, 261–275. doi: 10.1024/0044-3514.38.4.261

[ref34] Schneider B. (2001). Fits about bit. Appl. Psychol. Int. Rev. 50, 141–152. doi: 10.1111/1464-0597.00051

[ref35] Schroth H. (2019). Are you ready for gen Z in the workplace? Calif. Manag. Rev. 61, 5–18. doi: 10.1177/0008125619841006

[ref36] Schwartz S. H. (1992). Universals in the content and structure of values: theoretical advances and empirical tests in 20 countries. Adv. Exp. Soc. Psychol. 25, 1–65. doi: 10.1016/S0065-2601(08)60281-6

[ref37] Simms L. J. (2008). Classical and modern methods of psychological scale construction. Soc. Personal. Psychol. Compass 2, 414–433. doi: 10.1111/j.1751-9004.2007.00044.x

[ref38] Stiglbauer B. Batinic B. (2012). The role of Jahoda’s latent and financial benefits for work involvement: a longitudinal study. J. Vocat. Behav. 81, 259–268. doi: 10.1016/j.jvb.2012.07.008

[ref39] Tak J. (2011). Relationships between various person-environment fit types and employee withdrawal behavior: a longitudinal study. J. Vocat. Behav. 78, 315–320. doi: 10.1016/j.jvb.2010.11.006

[ref40] Tanwar K. Kumar A. (2019). Employer brand, person-organisation fit and employer of choice: investigating the moderating effect of social media. Pers. Rev. 48, 799–823. doi: 10.1108/PR-10-2017-0299

[ref41] Theurer C. P. Tumasjan A. Welpe I. M. Lievens F. (2018). Employer branding: a brand equity-based literature review and research agenda. Int. J. Manag. Rev. 20, 155–179. doi: 10.1111/ijmr.12121

[ref42] Twenge J. M. (2010). A review of the empirical evidence on generational differences in work attitudes. J. Bus. Psychol. 25, 201–210. doi: 10.1007/s10869-010-9165-6

[ref43] van den Broeck A. Ferris D. L. Chang C. H. Rosen C. C. (2016). A review of self-determination theory’s basic psychological needs at work. J. Manag. 42, 1195–1229. doi: 10.1177/0149206316632058

[ref44] van Vianen A. E. M. (2018). Person-environment fit: a review of its basic tenets. Annu. Rev. Organ. Psych. Organ. Behav. 5, 75–101. doi: 10.1146/annurev-orgpsych-032117-104702

[ref45] Westerman J. W. Cyr L. A. (2004). An integrative analysis of person-organization fit theories. Int. J. Sel. Assess. 12, 252–261. doi: 10.1111/j.0965-075X.2004.279_1.x

[ref46] Willner T. Lipshits-Braziler Y. Gati I. (2019). Construction and initial validation of the work orientation questionnaire. J. Career Assess. 28, 109–127. doi: 10.1177/1069072719830293

[ref47] Wrzesniewski A. McCauley C. Rozin P. Schwartz B. (1997). Jobs, careers, and callings: People’s relations to their work. J. Res. Pers. 31, 21–33. doi: 10.1006/jrpe.1997.2162

