# Supplementary material for: Work values across generations: Development of the New Work Values Scale (NWVS) and examination of generational differences
Source: Front Psychol. 2022 Nov 7;13:1028072. doi: 10.3389/fpsyg.2022.1028072 (PMC9677943; doi:10.3389/fpsyg.2022.1028072)
Supplement: Supplementary file 1 [file Data_Sheet_1.pdf]

Supplementary Material

Table A1. *Work Values Derived for the New Work Values Scales (NWVS) and Their Most Relevant Related Constructs.*

Focus	Work value	Definition	Most relevant related constructs
Sustainable organizational development	Readiness for Change	prefer organizations that do not persist in the old, but are open to innovations, consider change as an opportunity, and are ready to implement new ideas.	Tradition[a]
	Corporate Social Responsibility (CSR)	are looking for organizations that are well aware and take care of their economic, legal, ethical, and philanthropic responsibility.	Universalism[a]
	Inclusion	want organizations to especially care about fair and respectful treatment of all members and fight discrimination by all means.	Universalism[a]
Basic needs	Job Security	have a strong need for a secure workplace.	Security[a,] Need for Security[c]
	Participation	prefer a job where hierarchies are flat and everyone is welcome to contribute his or her own opinion and ideas.	Benevolence[a]
	Clarity	need structure, rules, and guidelines that provide stability, consistency, and orientation.	Conformity[a], Need for Clarity[c]
	Flexibility	want their job not to interfere with their personal lives.	Hedonism[a], Needs for Spatial & Temporal Flexibility and Segmentation[c]
Individual motivators	Money	are mostly motivated by monetary rewards.	Power[a], Job Orientation[b]
	Career	consider career development opportunities as very important to them.	Achievement and Power[a], Career Orientation[b], Need for Career Development[c]
	Development	want to always give their best and to therefore further develop their professional knowledge, skills, and competencies.	Achievement[a]. Career Orientation[b], Need for Efficiency[c]
	Stimulation	have a strong need for variety, challenges, and much going on in their job.	Stimulation[a], Busyness Orientation[b], Need for Challenge[c]
	Autonomy	want to design and do their work self-directedly.	Self-Direction[a], Need for Autonomy[c]
	Meaning	need their work to be meaningful and serve a collective purpose.	Calling Orientation[b]
	Relating	place great emphasis on good social relationships at work.	Social Embeddedness Orientation[b]
	Comfort[1]	*want their job to be a feel-good-place where they don't feel pressured or stressed.*	Hedonism[a]

[1] Comfort was not included in the final measurement instrument, as the data revealed substantial overlap with other work values.

[a]Schwartz Value Theory, [b]Willner and colleagues' Work Orientations, [c]Höge's Entreployee Work Orientations.

Table A2. *Items Included in the Validation Study.*

Work value	Item	English	German
Readiness for Change	RFC1*	A company should dare to sometimes try something new.	Ein Unternehmen sollte sich trauen, auch mal was Neues auszuprobieren.
	RFC2*	Innovative companies particularly appeal to me.	Innovative Unternehmen sprechen mich besonders an.
	RFC3	A company must not rely on old achievements, otherwise it will quickly be gone	Ein Unternehmen darf sich nicht auf alte Errungenschaften verlassen, sonst ist es schnell weg vom Fenster.
	RFC4	A company must constantly change in order to keep up with the market.	Ein Unternehmen muss sich ständig verändern, um am Markt mithalten zu können.
	RFC5	A company should always remember its tradition and continuously perfect the tried and tested.	Ein Unternehmen sollte sich stets auf seine Tradition besinnen und Bewährtes fortlaufend perfektionieren.
	RFC6	I am suspicious of companies that are always changing.	Unternehmen, die sich immer wieder verändern, sind mir suspekt.
Corporate Social Responsibility (CSR)	CSR1*	I would rather work for a company that helps make the world a better place.	Ich würde lieber für ein Unternehmen arbeiten, das dabei hilft, die Welt zu einem besseren Ort zu machen.
	CSR2*	Sustainability should be a key issue for all companies.	Nachhaltigkeit sollte ein zentrales Thema für alle Unternehmen sein.
	CSR3	The task of companies is more to secure their jobs rather than to improve the world.	Die Aufgabe von Unternehmen ist es eher seine Arbeitsplätze zu sichern als die Welt zu verbessern.
	CSR4	A company should be about profit first and foremost, after all, life is not a walk in the park.	In einem Unternehmen sollte es in erster Linie um Profit gehen, schließlich ist Arbeit kein Ponyhof.
	CSR5	Companies that advocate for the environment appeal to me.	Mich sprechen Unternehmen an, die sich für die Umwelt einsetzten.
	CSR6	Companies should also take on social responsibilities.	Unternehmen sollten auch gesellschaftliche Verantwortungen übernehmen.
Inclusion	INC1*	A company should put money into accessibility so that no one is discriminated against.	Ein Unternehmen sollte Geld in Barrierefreiheit stecken, damit niemand diskriminiert wird.
	INC2*	It is important to me that the proportion of women working there is taken into account in a company.	Es ist mir wichtig, dass in einem Unternehmen die Frauenquote berücksichtigt wird.
	INC3	Promotions should be based on qualification and performance.	Beförderungen sollten auf Basis von Qualifizierung und Leistung erfolgen.
	INC4	All employees should be able to speak at meetings.	Bei Besprechungen sollten alle Mitarbeiter/innen zu Wort kommen können.
	INC5	I think it is good if the salary depends on your own negotiating skills.	Ich finde es gut, wenn das Gehalt vom eigenen Verhandlungsgeschick abhängt.

	INC6	When I notice that colleagues are treated unfairly, it makes me angry.	Wenn ich merke, dass Kolleg/innen unfair behandelt werden, macht mich das wütend.
Security	SEC1*	A secure job is very important to me.	Mir ist ein sicherer Arbeitsplatz sehr wichtig.
	SEC2*	If I were to look for a new job, job security would be very important to me.	Wenn ich mir einen neuen Job suchen würde, wäre mir die Arbeitsplatzsicherheit sehr wichtig.
	SEC3	Exciting work is more important to me than a permanent employment contract.	Eine spannende Tätigkeit ist mir wichtiger als ein entfristeter Arbeitsvertrag.
	SEC4	I wish it were still like it used to be, where you worked for one employer for life.	Ich wünschte, es wäre noch wie früher, wo man ein Leben lang bei einem Arbeitgeber beschäftigt war.
	SEC5	In order to be able to plan personally, I need a secure job.	Um persönlich planen zu können, brauche ich einen sicheren Arbeitsplatz.
	SEC6	If I lost my job, it would not be so bad for me.	Würde ich meinen Job verlieren, wäre das für mich nicht so schlimm.
Participation	PAR1*	In a good company, all employees should have the opportunity to contribute ideas.	In einem guten Unternehmen sollten alle Mitarbeiter/innen die Möglichkeit haben, Ideen einzubringen.
	PAR2*	If employees are asked for their opinion and can have a say, that is a form of appreciation.	Wenn Mitarbeiter/innen nach ihrer Meinung gefragt werden und mitbestimmen können, ist das eine Form der Wertschätzung.
	PAR3	Flat hierarchies only lead to chaos.	Flache Hierarchien führen nur zu Chaos.
	PAR4	Ideally, managers always consult their staff when making important decisions.	Führungskräfte befragen im Idealfall immer ihre Mitarbeiter/innen bei wichtigen Entscheidungen.
	PAR5	Good work should be praised and note taken for granted.	Gute Arbeit sollte gelobt und nicht als selbstverständlich betrachtet werden.
	PAR6	Ideally, managers involve their employees in important decisions.	Im Idealfall beziehen Führungskräfte bei wichtigen Entscheidungen ihre Mitarbeiter/innen mit ein.
Clarity	CLA1*	A company should have clear structures.	Ein Unternehmen sollte klare Strukturen haben.
	CLA2*	I think employers should set clear rules that can be used as a guide.	Ich finde Arbeitgeber sollten klare Regeln aufstellen, an denen man sich orientieren kann.
	CLA3	A bit of chaos in the workplace encourages creative thinking.	Ein bisschen Chaos am Arbeitsplatz fördert das kreative Denken.
	CLA4	Clear guidelines give me stability and security.	Klare Vorgaben geben mir Halt und Sicherheit.
	CLA5	Constantly changing guidelines or processes make me restless.	Sich ständig ändernde Vorgaben oder Abläufe machen mich unruhig.
	CLA6	Guidelines and rules in a company only make work more difficult.	Vorgaben und Regeln in einem Unternehmen erschweren nur das Arbeiten.
Flexibility	FLE1*	I expect an employer to be understanding and flexible for unforeseeable private events.	Ich erwarte von einem/r Arbeitgeber/in Verständnis und Flexibilität für unvorhersehbare private Ereignisse.
	FLE2*	Work-life balance is very important to me.	Vereinbarkeit von Arbeit und Privatleben ist mir sehr wichtig.

	FLE3	It is important to me to be able to organize my working hours flexibly.	Es ist mir wichtig meine Arbeitszeiten flexibel gestalten zu können.
	FLE4	I am gladly available for my job in my free time as well.	Für meinen Job bin ich gerne auch in der Freizeit erreichbar.
	FLE5	I find it perfectly natural to answer work emails even when on holiday.	Ich finde es völlig selbstverständlich, auch im Urlaub Arbeitsemails zu beantworten.
	FLE6	After work, I do not want to have to deal with work anymore.	Nach Feierabend mag ich mich nicht mehr mit der Arbeit beschäftigen müssen.
Money	MON1*	A high salary is the most important thing to me.	Ein hohes Gehalt ist mir das Allerwichtigste.
	MON2*	I draw the greatest motivation for my work from a high salary.	Die größte Motivation für meine Arbeit schöpfe ich aus einem hohen Gehalt.
	MON3	Special achievements should be remunerated with bonus payments.	Besondere Leistungen sollten mit Bonuszahlungen vergütet werden.
	MON4	Exciting work is more important to me than a good salary.	Eine spannende Tätigkeit ist mir wichtiger als ein gutes Gehalt.
	MON5	For me, salary is the most important form of recognition.	Gehalt ist für mich die wichtigste Form der Anerkennung.
	MON6	I would also work for little money if I could learn something in the process.	Ich würde auch für wenig Geld arbeiten, wenn ich etwas dabei lernen kann.
	MON7	If the social benefits and overall conditions are right, I could imagine adjusting my salary expectations downwards.	Wenn die Sozialleistungen und die Rahmenbedingungen passen, könnte ich mir vorstellen meine Gehaltsvorstellungen nach unten anzupassen.
Career	CAR1*	Opportunities for advancement motivate me.	Aufstiegsmöglichkeiten motivieren mich.
	CAR2*	I want to make a career in my job.	Ich will in meinem Job Karriere machen.
	CAR3	A good company should offer sufficient opportunities for advancement.	Ein gutes Unternehmen sollte ausreichend Aufstiegsmöglichkeiten bieten.
	CAR4	I want to go far in my career.	Ich möchte es beruflich weit bringen.
	CAR5	I spare no effort to achieve a lot professionally.	Ich scheue keine Mühen, um beruflich viel zu erreichen.
	CAR6	I am looking for professional competitive situations to prove myself.	Ich suche beruflich Konkurrenzsituationen, um mich zu beweisen.
	CAR7	In my job, I do not just want to follow instructions, I want to have an influence myself.	In meinem Beruf möchte ich nicht nur Anweisungen befolgen, sondern selbst Einfluss haben.
Development	DEV1*	Further training is important to me.	Fortbildungen sind mir wichtig.
	DEV2*	In my job, I always want to develop myself and my knowledge.	Im Job will ich mich und meine Kenntnisse stets weiterentwickeln.
	DEV3	I am only satisfied with my professional performance when I feel I have given my best.	Ich bin mit meiner beruflichen Leistung erst zufrieden, wenn ich das Gefühl habe mein Maximum gegeben zu haben.

	DEV4	I am always striving to develop professionally.	Ich bin stets darum bemüht mich beruflich weiterzuentwickeln.
	DEV5	I spare no effort to expand my knowledge and skills.	Ich scheue keine Mühen, mein Wissen und meine Kompetenzen zu erweitern.
	DEV6	When I do something, I always do it in a way that I can be proud of.	Wenn ich etwas erledige, dann immer so, dass ich stolz darauf sein kann.
Stimulation	STI1*	There has to be something going on at my work before I feel good.	Bei meiner Arbeit muss was los sein, dann fühle ich mich erst wohl.
	STI2*	I really enjoy a job when I am always faced with new challenges.	Ein Job macht mir dann wirklich Freude, wenn ich immer wieder vor neuen Herausforderungen stehe.
	STI3	I see challenges as opportunities rather than obstacles.	Herausforderungen sehe ich eher als Chance und nicht als Hindernis.
	STI4	I need a lot of different things to do, otherwise I get bored quickly.	Ich brauche viel Unterschiedliches zu tun, sonst wird mir schnell langweilig.
	STI5	I enjoy new challenges.	Ich habe Freude an neuen Herausforderungen.
	STI6	I like to have different work assignments.	Ich habe gerne verschiedene Arbeitsaufgaben.
	STI7	I like to look for challenging tasks at work.	Ich suche mir in der Arbeit gerne anspruchsvolle Aufgaben.
Autonomy	AUT1*	I prefer to design my work completely freely according to my own specifications.	Ich gestalte meine Arbeit am liebsten völlig frei nach meinen eigenen Vorgaben.
	AUT2*	In my job, it is important for me to be able to decide for myself when I do which activity.	Im Job ist es mir wichtig, selbst bestimmen zu können, wann ich welche Tätigkeit durchführe.
	AUT3	Good managers give their employees as much freedom of choice as possible.	Gute Führungskräfte lassen ihren Mitarbeiter/innen möglichst viel Entscheidungsfreiheit.
	AUT4	I am happy when I do not have to make as many decisions at work.	Ich bin froh, wenn ich in der Arbeit wenig Entscheidungen treffen muss.
	AUT5	I do **not** want to have to check up with a superior for every decision.	Ich möchte **nicht** jede Entscheidung mit einem/r Vorgesetzten abklären müssen.
	AUT6	I want to make decisions independently.	Ich möchte selbstständig Entscheidungen treffen.
Meaning	MEA1*	I want to do something good for others with my work.	Ich möchte mit meiner Arbeit etwas Gutes für andere bewirken.
	MEA2*	With my work, I want to contribute to making the world a better place.	Mit meiner Arbeit möchte ich dazu beitragen, die Welt zu verbessern.
	MEA3	The feeling of contributing something important to the common good is the driving force behind my work.	Das Gefühl etwas Wichtiges zum Allgemeinwohl beizutragen, ist der Motor für meine Arbeit.
	MEA4	Money alone is **not** enough for me to find meaning in my work.	Geld alleine reicht mir bei meiner Arbeit **nicht** zur Sinnstiftung.
	MEA5	With my work, I want to be able to make a difference and make the world a little better.	Mit meiner Arbeit möchte ich etwas bewirken können und die Welt ein Stück besser machen.

	MEA6	It makes me proud, when I notice that my work has made a difference.	Wenn ich merke, dass meine Arbeit etwas bewirkt hat, macht mich das stolz.
Relating	REL1*	It is important for me to be friends with my colleagues.	Mir ist es wichtig mit meinen Kolleg/innen auch befreundet zu sein.
	REL2*	Regular social events alongside work are a sign of a good company.	Regelmäßige soziale Veranstaltungen neben der Arbeit zeichnen ein gutes Unternehmen aus.
	REL3	A friendly working atmosphere jeopardizes productivity.	Ein freundschaftliches Arbeitsklima gefährdet die Produktivität.
	REL4	A good work team should always be friendly with each other.	Ein gutes Arbeitsteam sollte stets freundschaftlich miteinander umgehen.
	REL5	Shared lunch breaks promote a good working atmosphere.	Gemeinsame Mittagspausen fördern ein gutes Arbeitsklima.
	REL6	I think team-building measures are a waste of time.	Ich halte Teambuilding-Maßnahmen für Zeitverschwendung.
	REL7	I would like to be able to talk about private things with colleagues at work.	Ich möchte mich mit Arbeitskolleg/innen gerne auch mal über Privates austauschen können.
Comfort	COM1	Work does **not** necessarily have to be fun.	Arbeit muss **nicht** unbedingt Spaß machen.
	COM2	You also have to feel comfortable at work.	Bei der Arbeit muss man sich auch wohl fühlen.
	COM3	Work should be a place of well-being for me.	Die Arbeit soll für mich ein Wohlfühlort sein.
	COM4	It stresses me out when colleagues work at a fast pace.	Es stresst mich, wenn Kolleg/innen ein schnelles Arbeitstempo vorlegen.
	COM5	I like to work under pressure.	Ich arbeite gerne unter Druck.
	COM6	I make mistakes under pressure, so my employer should never stress me out.	Unter Druck mache ich Fehler, deswegen sollte mein/e Arbeitgeber/in mich nie stressen.

Note. * = included in the final measure.

Table A3. *Bivariate Correlations (*n *= 316) of the New Work Values Scale with Willner and Colleagues' (2019) Work Orientation Questionnaire, Höge's (2011) Entreployee Work Orientation Scales, and the German 21-Item Version of the Portraits Value Questionnaire (PVQ; Schmidt et al., 2007).*

Scales (Cronbach's α reliability estimates)	RFC	CSR	INC	SEC	PAR	CLA	FLE	MON	CAR	DEV	STI	AUT	MEA	REL
**Work Orientation Questionnaire**														
Job (.74)	.06	-.03	-.01	.16	.13	.14	.23	.35	-.02	-.15	-.08	.02	-.12	.06
Career (.92)	.26	-.01	.13	.06	-.01	.05	.14	.26	.79	.52	.37	.21	.17	.25
Calling (.76)	.13	.19	.21	.05	-.01	.09	-.03	-.03	.31	.47	.39	.15	.49	.25
Busyness (.80)	.07	.04	.11	.02	-.02	-.02	-.14	-.02	.28	.29	.33	-.04	.15	.18
Social Embedded-ness (.82)	.18	.10	.19	.24	.15	.12	.25	.01	.34	.41	.30	.19	.37	.59
**Entreployee Work Orientation Scales**														
Need for …														
Efficiency (.80)	.38	.23	.30	.27	.18	.31	.18	.04	.46	.56	.51	.26	.44	.27
Challenge (.86)	.28	.16	.19	.08	.02	.17	.14	.00	.46	.52	.69	.22	.32	.24
Clarity (.76)	.23	.09	.17	.16	.08	.30	.18	.16	.14	.05	.14	-.05	.10	.22
Career Develop-ment (.84)	.38	.13	.23	.10	.07	.11	.29	.23	.61	.54	.46	.29	.26	.27
Autonomy (.79)	.31	.13	.17	.05	.19	.16	.35	.03	.33	.41	.40	.59	.29	.17
Security (.74)	.09	.08	.18	.46	.16	.30	.16	.13	.06	.18	.11	.19	.22	.27
Spatial Flexibility (.65)	.24	.21	.17	-.03	.05	.03	.49	.03	.14	.18	.26	.39	.25	.11
Temporal Flexibility (.86)	.24	.15	.09	.01	.12	.04	.66	.05	.21	.23	.22	.47	.24	.14
Segmentation (.85)	.10	.13	.10	.14	.17	.23	.30	.12	.03	.02	-.03	.10	.09	.10
**PVQ**														
Self-Direction (.57)	-.19	-.20	-.12	.00	-.20	-.12	-.16	.03	-.14	-.25	-.24	-.36	-.14	-.05
Power (.46)	-.22	.08	.01	-.07	-.01	-.07	-.08	-.39	-.41	-.21	-.17	-.22	-.03	-.12
Universalism (.67)	-.15	-.53	-.39	-.10	-.21	-.05	-.10	.13	.00	-.24	-.14	-.18	-.42	-.09
Achievement (.73)	-.19	-.03	-.01	-.12	-.07	-.11	-.07	-.25	-.47	-.32	-.20	-.14	-.13	-.15
Security (.59)	.00	-.04	-.07	-.45	-.06	-.17	-.12	-.19	-.05	-.05	.04	-.13	-.11	-.16
Stimulation (.79)	-.18	-.15	-.07	.15	-.01	.06	-.03	-.05	-.36	-.34	-.40	-.13	-.18	-.18
Conformity (.50)	-.05	.00	-.04	-.24	-.04	-.09	-.03	-.16	-.10	-.07	.00	-.07	-.14	-.11
Tradition (.37)	-.01	-.06	-.06	-.23	-.01	-.17	-.16	-.07	-.08	-.05	-.01	-.06	-.11	-.11
Hedonism (.77)	-.23	-.10	-.07	-.10	-.09	-.07	-.06	-.14	-.31	-.22	-.26	-.14	-.09	-.12
Benevolence (.61)	-.20	-.30	-.28	-.17	-.24	-.17	-.10	.06	-.07	-.21	-.16	-.23	-.31	-.10

Note. $r > |.11|$ are significant at $p < .05$.
RFC = Readiness for Change, CSR = Corporate Social Resonsibility, INC = Inclusion, SEC = Security, PAR = Participation, CLA = Clarity, FLE = Flexibility, MON = Money, CAR = Career, DEV = Development, STI = Stimulation, AUT = Autonomy, MEA = Meaning, REL = Relating.

Table A4. *Items Included in the Final Version of the New Work Values Scale (NWVS).*

Work value	Item	English	German
Readiness for Chage	RFC1	A company should dare to sometimes try something new.	Ein Unternehmen sollte sich trauen, auch mal was Neues auszuprobieren.
	RFC2	Innovative companies particularly appeal to me.	Innovative Unternehmen sprechen mich besonders an.
Corporate Social Responsibility (CSR)	CSR1	I would rather work for a company that helps make the world a better place.	Ich würde lieber für ein Unternehmen arbeiten, das dabei hilft, die Welt zu einem besseren Ort zu machen.
	CSR2	Sustainability should be a key issue for all companies.	Nachhaltigkeit sollte ein zentrales Thema für alle Unternehmen sein.
Inclusion	INC1	A company should put money into accessibility so that no one is discriminated against.	Ein Unternehmen sollte Geld in Barrierefreiheit stecken, damit niemand diskriminiert wird.
	INC2	It is important to me that the proportion of women working there is taken into account in a company.	Es ist mir wichtig, dass in einem Unternehmen die Frauenquote berücksichtigt wird.
Security	SEC1	A secure job is very important to me.	Mir ist ein sicherer Arbeitsplatz sehr wichtig.
	SEC2	If I were to look for a new job, job security would be very important to me.	Wenn ich mir einen neuen Job suchen würde, wäre mir die Arbeitsplatzsicherheit sehr wichtig.
Participation	PAR1	In a good company, all employees should have the opportunity to contribute ideas.	In einem guten Unternehmen sollten alle Mitarbeiter/innen die Möglichkeit haben, Ideen einzubringen.
	PAR2	If employees are asked for their opinion and can have a say, that is a form of appreciation.	Wenn Mitarbeiter/innen nach ihrer Meinung gefragt werden und mitbestimmen können, ist das eine Form der Wertschätzung.
Clarity	CLA1	A company should have clear structures.	Ein Unternehmen sollte klare Strukturen haben.
	CLA2	I think employers should set clear rules that can be used as a guide.	Ich finde Arbeitgeber sollten klare Regeln aufstellen, an denen man sich orientieren kann.
Flexibility	FLE1	I expect an employer to be understanding and flexible for unforeseeable private events.	Ich erwarte von einem/r Arbeitgeber/in Verständnis und Flexibilität für unvorhersehbare private Ereignisse.
	FLE2	Work-life balance is very important to me.	Vereinbarkeit von Arbeit und Privatleben ist mir sehr wichtig.
Money	MON1	A high salary is the most important thing to me.	Ein hohes Gehalt ist mir das Allerwichtigste.
	MON2	I draw the greatest motivation for my work from a high salary.	Die größte Motivation für meine Arbeit schöpfe ich aus einem hohen Gehalt.
Career	CAR1	Opportunities for advancement motivate me.	Aufstiegsmöglichkeiten motivieren mich.
	CAR2	I want to make a career in my job.	Ich will in meinem Job Karriere machen.

Development	DEV1	Further training is important to me.	Fortbildungen sind mir wichtig.
	DEV2	In my job, I always want to develop myself and my knowledge.	Im Job will ich mich und meine Kenntnisse stets weiterentwickeln.
Stimulation	STI1	There has to be something going on at my work before I feel good.	Bei meiner Arbeit muss was los sein, dann fühle ich mich erst wohl.
	STI2	I really enjoy a job when I am always faced with new challenges.	Ein Job macht mir dann wirklich Freude, wenn ich immer wieder vor neuen Herausforderungen stehe.
Autonomy	AUT1	I prefer to design my work completely freely according to my own specifications.	Ich gestalte meine Arbeit am liebsten völlig frei nach meinen eigenen Vorgaben.
	AUT2	In my job, it is important for me to be able to decide for myself when I do which activity.	Im Job ist es mir wichtig, selbst bestimmen zu können, wann ich welche Tätigkeit durchführe.
Meaning	MEA1	I want to do something good for others with my work.	Ich möchte mit meiner Arbeit etwas Gutes für andere bewirken.
	MEA2	With my work, I want to contribute to making the world a better place.	Mit meiner Arbeit möchte ich dazu beitragen, die Welt zu verbessern.
Relating	REL1	It is important for me to be friends with my colleagues.	Mir ist es wichtig mit meinen Kolleg/innen auch befreundet zu sein.
	REL2	Regular social events alongside work are a sign of a good company.	Regelmäßige soziale Veranstaltungen neben der Arbeit zeichnen ein gutes Unternehmen aus.

Note. Response Format 1 = *do not agree at all*, 3 = *part-part*, and 5 = *fully agree*.
German Response Format 1 = *stimme überhaupt nicht zu*, 3 = *teils-teils*, 5 = *stimme voll und ganz zu*.

Table A5. *Factor Loadings (CFA) and Spearman Brown Reliability Estimates of the New Work Values Scale (Study 2,* n *= 956).*

Work value	Item	Factor loading	Reliability
Readiness for Change	RFC1	.75	.73
	RFC2	.77	
Corporate Social Responsibility	CSR1	.75	.76
	CSR2	.83	
Inclusion	INC1	.81	.67
	INC2	.63	
Security	SEC1	.88	.87
	SEC2	.87	
Participation	PAR1	.82	.80
	PAR2	.81	
Clarity	CLA1	.86	.79
	CLA2	.76	
Flexibility	FLE1	.66	.68
	FLE2	.78	
Money	MON1	.86	.84
	MON2	.84	
Career	CAR1	.84	.83
	CAR2	.84	
Development	DEV1	.80	.82
	DEV2	.87	
Stimulation	STI1	.65	.71
	STI2	.85	
Autonomy	AUT1	.74	.76
	AUT2	.83	
Meaning	MEA1	.79	.83
	MEA2	.89	
Relating	REL1	.72	.70
	REL2	.75	

Table A6. *Inter-Correlations of the New Work Values Scale and Socio-Demographics.*

Variable	1a	1b	1c	2	3	4a	4b	4c	4d	5	6	7	8	9	10	11	12	13	14	15	16	17	18
1 Gender:																							
1a Female				-.16	-.07	-.18	.06	.12	.12	-.03	.10	**.13**	.06	.10	.02	-.02	**-.14**	-.07	.11	.08	.01	**.20**	.07
1b Male				.15	.08	.19	-.07	-.12	-.12	.04	-.10	**-.14**	-.06	-.10	-.01	.02	**.14**	.07	-.10	-.07	.00	**-.20**	-.07
1c Diverse				.03	-.03	-.08	.13	-.02	-.02	-.07	-.02	.04	.00	-.05	-.04	-.02	.00	-.02	-.06	-.06	-.05	.00	.02
2 Age	-.36	.36	-.02		-.16	.03	.22	.21	-.53	-.08	-.01	.00	.03	.03	.07	**-.12**	-.08	**-.32**	**-.20**	-.11	.03	-.08	**-.17**
3 Education[1]	-.01	.01	.00	-.18		.06	-.13	-.05	.12	.00	.10	-.07	**-.24**	**-.18**	-.07	.08	-.02	.08	.10	.10	**.13**	-.02	-.14
4 Employment status:																							
4a Employed	-.18	.17	.04	.30	.06					.00	-.07	-.09	.03	-.02	.09	.04	**.12**	-.04	-.04	.06	.04	-.10	.01
4b Unemployed	-.13	.13	-.02	.14	-.15					-.03	.03	.05	-.01	.04	-.09	-.08	-.09	-.05	-.09	-.10	-.06	.04	-.04
4c OLF	.17	-.17	-.02	-.03	-.06					.00	.01	.01	.03	-.01	.02	-.07	-.09	-.10	.00	-.05	-.07	.03	-.02
4d In education	.22	-.22	-.02	-.51	.09					.04	.06	.08	-.06	.00	-.05	.09	.00	**.22**	**.19**	.08	.06	.10	.06
5 RFC	-.05	.05	-.01	.00	**.08**	.04	**-.07**	-.03	.03		.30	.29	.09	.30	.19	.25	.07	.30	.31	.29	.21	.16	.21
6 CSR	**.10**	**-.10**	-.01	**-.09**	**.07**	-.02	**-.07**	.00	**.08**	.35		.42	.04	.33	.09	.18	-.10	.02	.23	.18	.11	.49	.20
7 INC	**.16**	**-.16**	.02	**-.12**	-.01	**-.07**	-.05	.02	**.12**	.27	.56		.16	.21	.10	.16	.07	.13	.20	.16	.13	.40	.28
8 SEC	**.07**	**-.07**	.01	.04	**-.11**	**.08**	.00	-.01	**-.10**	.15	.14	.16		.21	.24	.07	.18	.14	.12	.04	.04	.08	.24
9 PAR	.05	-.05	-.02	**.07**	.01	**.07**	-.03	.00	**-.07**	.40	.37	.30	.40		.15	.21	-.01	.06	.12	.08	.16	.20	.18
10 CLA	-.04	.03	.04	**.18**	**-.09**	**.14**	-.01	-.03	**-.16**	.24	.17	.15	.45	.39		.11	.11	.11	.18	.14	.01	.13	.04
11 FLE	**.07**	**-.07**	.01	-.03	.06	.02	-.03	.02	-.02	.25	.28	.21	.31	.48	.28		.02	.18	.19	.13	.39	.18	.30
12 MON	-.06	.06	-.01	**-.12**	**-.07**	.02	-.01	-.02	.00	.11	-.10	.01	.17	-.02	.13	.05		.30	.06	.05	.14	-.04	.08
13 CAR	.01	-.02	.03	**-.29**	**.11**	-.03	-.04	-.08	**.14**	.29	.15	.22	.12	.13	.13	.10	.36		.57	.44	.27	.17	.31
14 DEV	.04	-.05	.06	**-.11**	**.10**	-.01	**-.07**	-.03	**.09**	.32	.27	.32	.10	.24	.20	.12	.04	.51		.47	.26	.36	.31
15 STI	-.06	.06	-.01	**-.07**	.03	**.08**	**-.07**	-.03	-.02	.31	.23	.19	.12	.22	.15	.09	.11	.42	.40		.31	.30	.25
16 AUT	**-.10**	**.10**	-.02	.06	**.18**	**.19**	**-.11**	-.03	**-.15**	.19	.09	-.03	.06	.23	.11	.31	.15	.20	.12	.24		.23	.16
17 MEA	**.07**	**-.08**	.02	**-.09**	**.08**	.02	**-.09**	-.04	**.08**	.24	.50	.44	.11	.29	.19	.20	-.03	.30	.38	.33	.13		.32
18 REL	.06	-.05	-.02	**-.11**	-.05	.02	**-.07**	-.02	.05	.20	.17	.31	.16	.24	.21	.17	.22	.33	.32	.27	.08	.30	

Note. Study 1 ($n = 316$) above the Diagonal, $r > |.11|$ are significant at $p < .05$. Study 2 ($n = 956$) below the Diagonal, $r > |.06|$ are significant at $p < .05$. RFC = Readiness for Change, CSR = Corporate Social Resonsibility, INC = Inclusion, SEC = Security, PAR = Participation, CLA = Clarity, FLE = Flexibility, MON = Money, CAR = Career, DEV = Development, STI = Stimulation, AUT = Autonomy, MEA = Meaning, REL = Relating.
[1] Education: 1 = Compulsory, 2 = Vocational, 3 = High School, 4 = University.
